# NMD-12: A new machine-learning derived screening instrument to detect mild cognitive impairment and dementia

**DOI:** 10.1371/journal.pone.0213430

**Published:** 2019-03-08

**Authors:** Pai-Yi Chiu, Haipeng Tang, Cheng-Yu Wei, Chaoyang Zhang, Guang-Uei Hung, Weihua Zhou

**Affiliations:** 1 Department of Neurology, Show Chwan Memorial Hospital, Changhua, Taiwan; 2 School of Computing, University of Southern Mississippi, Hattiesburg, MS, United States of America; 3 Department of Neurology, Chang Bing Show Chwan Memorial Hospital, Changhua, Taiwan; 4 Department of Exercise and Health Promotion, College of Education, Chinese Culture University, Taipei, Taiwan; 5 Department of Nuclear Medicine, Chang Bing Show Chwan Memorial Hospital, Changhua, Taiwan; University of New South Wales, AUSTRALIA

## Abstract

**Introduction:**

Using machine learning techniques, we developed a brief questionnaire to aid neurologists and neuropsychologists in the screening of mild cognitive impairment (MCI) and dementia.

**Methods:**

With the reduction of the survey size as a goal of this research, feature selection based on information gain was performed to rank the contribution of the 45 items corresponding to patient responses to the specified questions. The most important items were used to build the optimal screening model based on the accuracy, practicality, and interpretability. The diagnostic accuracy for discriminating normal cognition (NC), MCI, very mild dementia (VMD) and dementia was validated in the test group.

**Results:**

The screening model (NMD-12) was constructed with the 12 items that were ranked the highest in feature selection. The receiver-operator characteristic (ROC) analysis showed that the area under the curve (AUC) in the test group was 0.94 for discriminating NC vs. MCI, 0.88 for MCI vs. VMD, 0.97 for MCI vs. dementia, and 0.96 for VMD vs. dementia, respectively.

**Discussion:**

The NMD-12 model has been developed and validated in this study. It provides healthcare professionals with a simple and practical screening tool which accurately differentiates NC, MCI, VMD, and dementia.

## Introduction

Current screening tools for dementia are intended for detecting of early cognitive impairment and distinguishing these patients from the normal cognitive population [1–6]. These kinds of tools are commonly used to screen patients with mild cognitive impairment (MCI) or dementia in community- or hospital-based settings. Several limitations have been reported with respect to these screening tools. When applied to populations with different cultures and languages, a relatively low sensitivity [7] or specificity [8] may bias the test; these tools cannot accurately differentiate MCI from dementia; the cutoff scores vary across different countries or cultures [1–5,8,9]. More importantly, identifying cutoff scores in cognitive screening test such as Montreal Cognitive Assessment (MoCA) [10,11] or Cognitive Abilities Screening Instrument (CASI) [12,13] is even more challenging for clinical application. The information acquisition may be different from participants or from informants.

Even if these issues are resolved when using the existing screening tools, there are still a lot of challenges in their clinical applications. Major issues lie in the early detection of individuals with cognitive impairment as well as the development of further prevention or management strategies. To improve the diagnostic accuracy and extend appropriate populations with different types and stages of dementia, there is a demand to develop new screening tools. With the available feature selection methods in machines learning (ML), it is possible for us to determine the weights of features, discard the insignificant ones and reduce the complexity of our screening task. Therefore, the aim of this study was to use ML to develop a brief and accurate informant-based questionnaire for the screening of mild cognitive impairment (MCI) and dementia due to Alzheimer’s disease (AD) and other diseases.

## Methods

### Study population

This is a sub-study of the “history-based artificial intelligent clinical dementia diagnostic system (HAICDDS) project”. Before this study, A preliminary study to test the appropriateness of the HAICDDS was done with the approval (Show Chwan IRB number: 1041208) by the Medical Research Ethics Committee of Show Chwan Memorial Hospital. In the preliminary study, 120 participants and their informants signed inform consents and completed the study. With appropriateness of the preliminary study, the project is currently taking place in three centers of the Show Chwan Healthcare System (two in central Taiwan and one in southern Taiwan). In this project, we consecutively enrolled and selected 1,354 individuals aged from 40–100 years with normal cognition (NC), MCI, or dementia. The subjects were then randomly divided into the training group (716 subjects), which was used to build the NMD questionnaire, and the test group (638 subjects), which was used to validate the NMD questionnaire in discriminating NC, MCI, very mild dementia (VMD) and dementia. To protect the right of each participant, especially when part of the participants presented with mild deterioration of mentality and might be in the MCI or VMD stage, all participants and their informants received a thorough explanation of the purpose and a possibility of the further application of the data. Oral agreement of the participants and their informants were obtained before their participating in the project.

In the HAICDDS project, we developed an instrument based on a structured 45-item questionnaire for the clinical diagnosis of the severity of dementia or cognitive impairment. The questionnaire was composed of 12 memory, 5 orientation/visuospatial functions, 6 judgment/executive functions, 8 languages, 9 instrumental activities of daily living, and 5 basic activities of daily living questions. Informants of the participants were interviewed by well-trained neuropsychologists with continuous quality control to achieve necessary quality standards. Twenty-six patients were selected before the training period to obtain the inter-rater reliability of the structured 45-item questionnaire and the results revealed a good intra-class correlation coefficient of 0.830. The final diagnosis of the subtype of dementia was made in a consensus meeting that was composed of neurologists and neuropsychologists. The severity was graded according to the staging of CDR. This study was performed in accordance with the Declaration of Helsinki. The participants were selected from the register-based database of the Show Chwan Health System. The study design was retrospective, and the data were analyzed anonymously. The Medical Research Ethics Committee of Show Chwan Memorial Hospital reviewed the project, and the Data Inspectorate approved the study.

### Definition of normal cognition (NC), MCI, VMD, dementia, or cognitive impairment (CI)

NC referred to individuals who did not meet criteria for any of the conditions listed in the National Institute on Aging-Alzheimer’s Association (NIA-AA) core clinical criteria for all-cause dementia [14] and had a CDR score of 0 [15].

MCI was defined as the individuals who had cognitive change with impairment in the domains of orientation and/or judgment but without impairment in social or occupational functioning and had a CDR score of 0.5 [16]. In addition, at least one cognitive domain in CASI adjusted with age and education level should be impaired [12,13]. In the domains of community affairs, home hobbies and personal care, the CDR should be 0.

VMD was defined as the individuals who met the NIA-AA criteria for all-cause dementia with a CDR score of 0.5 [14], had mild impairment in 2 or more cognitive domains and had mild decline in daily functions, including the domains of community affairs, home hobbies or personal care in which the CDR should be ≧0.5.

The definition of all-cause dementia was based on the core clinical criteria recommended by the NIA-AA [14]. The different types of dementia were diagnosed according to each consensus criteria. A structured clinical history was taken from the participant and the principal caregiver. The clinical history was taken to detect any subtle change of behavior or personality and any mental decline from previous levels of functioning, and to determine whether this decline interfered with the ability to function at work or in routine activities. The cognitive impairment could not be explained by delirium and major psychiatric disorders. In addition to the history of cognitive status, objective assessments including the CDR, MMSE, CASI, and MoCA were performed to evaluate memory, executive function, orientation, visual-spatial ability, and language function. The severity of dementia was then determined by the CDR. Daily function was assessed with Instrumental Activities of Daily Living (IADL) scale [17]. Neuropsychiatric Inventory (NPI) was used to assess the neuropsychiatric symptoms of participants [18].

CI was defined as the individuals who did not meet criteria of MCI, VMD, or dementia.

### Machine learning to build an NMD questionnaire

In this study, we use machine learning methods to determine the weights of features in the training dataset and reduce the dimensionality of the data by selecting the top-ranked features. The 45 items in the questionnaire were treated as features and each of them has different importance in the prediction of dementia diagnosis. Removing those redundant or unnecessary features with low importance can simplify the procedure of diagnosis, enhance the practicality in clinic, and may increase the accuracy of the diagnosis. Automated feature selection by information gain (IG) ranking in ML was used to rank the importance of all 45 features, and then the low ranking features were filtered out. The IG method is briefly described as follows.

IG measures the classification effectiveness of a feature based on entropy, a notation in information theory, which can be applied to evaluate the importance of features [19–20]. The IG of a feature is to measure the entropy difference of a system when including and excluding this feature, as shown in Eqs (1)–(3).

$$E(D)=-\sum_{i=0}^{m}p_{i}{log}_{2}(p_{i})$$ (1)

$$E_{F}(D)=-\sum_{j=1}^{v}\left({|D}_{i}|/|D|)\times E(D_{j}\right)$$ (2)

$${IG=E(D)-E}_{F}(D)$$ (3)

where *D* is the data sample in training set, *F* is a feature, m is the number of possible outcomes (or classes), and *p*_*i*_ is the nonzero probability that an arbitrary tuple in *D* belongs to class *C*_*i*_. *D*_*j*_ is a subset of *D* containing distinct values of *F*, and *v* is the number of distinct values in *F*. Let *C*_*i*,*D*_ be the set of records of class *C*_*i*_ and |*C*_*i*,*D*_| and |*D*| denote the number of medical records in the *C*_*i*,*D*_ and *D*, respectively. *p*_*i*_ can be estimated by |*C*_*i*,*D*_|/|*D*|. Eq (1) calculates the entropy *E(D)* of *D* and it represents the information needed to classify a tuple in the training data. Eq (2) computes *E*_*F*_*(D)* that denotes the amount of information required to arrive at an exact classification based on the partition by feature *F*. The *IG* measurement of a feature is defined as the difference between *E(D)* of classes and *E*_*F*_*(D)*, as given in Eq (3). The features with higher *IG* values are considered more important than those with lower *IG*. Therefore, the importance of all 45 features was ranked based on the *IG* values.

This feature selection method based on information gain was implemented in Weka [19] that is an open-source ML toolkit for knowledge analysis. It was used in our study to rank select the top 12 features from a total of 45 features.

In the training group, 24 of the 45 features had IG > 0 in the diagnosis of NC, MCI, and dementia, so they were selected for the further model refinement. Among these 24 items, 8Q (the first 8 items with the highest information gain), 9Q, 10Q, 12Q, 14Q, and 16Q were compared by the ROC curve analysis to find the briefest screening instrument that could accurately distinguish NC, MCI, and dementia. Another twenty-eight patients were selected before the training period to obtain the inter-rater reliability of the NMD-12 questionnaire and the results revealed a good intra-class correlation coefficient of 0.870.

The diagnostic accuracy of the optimal questionnaire for discriminating NC, MCI, VMD and dementia was further validated in the test group which consisted of 638 subjects. The Chinese version of SPSS 19.0 for Windows (IBM, SPSS Inc., Chicago) was used for statistical analyses. Comparisons between different groups on demographic data, neuropsychological tests, a total score of IADL, AD8, our optimal questionnaire, and the composite scores of NPI were analyzed using one-way ANOVA. Gender was analyzed with the chi-square test. We used data from two-by-two tables to calculate the sensitivity and specificity as well as the area under the curve (AUC) from ROC curves. Cut-off values were the point on the curve with minimum distance from the left-upper corner of the unit square. The significance level was set at p< 0.05 for all tests.

## Results

12Q (the first 12 items with the highest information gain) was selected as the optimal version of NMD (NMD-12) based on accuracy and practicality. NMD-12 is composed of questionnaires which assesses memory (4Q), orientation (2Q), judgment (2Q), community affair (3Q), and home hobbies (1Q). The detailed information of NMD-12 questionnaire is shown in S1 Appendix with original Chinese printing and tentative English translation. The clinical interpretability of NMD-12 was also confirmed by the neurologists and neuropsychologists who participated in this project.

In the test group with 638 participants, there were 53 NC (CDR = 0), 91 MCI (CDR = 0.5), 108 VMD (CDR = 0.5), and 386 dementia (CDR > = 1). Among the 494 patients with VMD or dementia, 202 (40.9%) were Alzheimer’s disease (AD), followed by vascular dementia (138, 27.9%), dementia with Lewy bodies (64, 12.9%), Parkinson’s disease dementia (36, 7.3%), other/undetermined dementia (24, 4.9%), normal pressure hydrocephalus (21, 4.3%), and frontotemporal dementia (9, 1.8%). Detailed demographical data were given in Table 1 and showed the dysfunction of cognition and activities of daily living as well as the severity of neuropsychiatric symptoms deteriorated as the stages of dementia increased.

**Table 1:** Comparison of demographic data among the groups with different stages of cognitive impairment.

Group	CDR 0	CDR 0.5 (MCI)	CDR 0.5 (VMD)	CDR≧1	F/x^2^	*p*
**N**	53	91	108	386		
**Age, year (SD, range)**	67.8 (10.7, 43–92)	71.4 (9.3, 40–90)	76.1 (8.8, 51–99)	79.5 (8.6, 44–99)	41.533	< 0.001*
**Female N (%)**	25 (47.2)	46 (50.5)	65 (60.2)	226 (58.5)	4.026	0.259
**Education, year (SD, range)**	6.9 (5.1, 0–18)	6.0 (4.3, 0–18)	4.7 (4.2, 0–18)	4.1 (4.5, 0–24)	8.929	< 0.001**
**NMDQ-12 (SD, range)**	0.6 (0.7, 0–3)	3.1 (1.7, 0–10)	6.4 (2.1, 2–11)	11.2 (1.3, 4–12)	1323.355	< 0.001*
**AD8 (SD, range)**	0.6 (0.7, 0–3)	2.5 (1.1, 0–6)	4.1 (1.4, 1–7)	6.6 (0.9, 3–8)	871.829	< 0.001*
**MMSE (SD, range)**	26.0 (4.1, 14–30)	24.1 (4.0, 14–30)	19.7 (4.7, 8–29)	11.7 (6.4, 0–27)	206.537	< 0.001*
**MoCA (SD, range)**	21.3 (7.0, 8–30)	17.6 (5.9, 3–30)	12.4 (6.0, 1–27)	5.7 (4.7, 0–22)	229.791	< 0.001*
**CASI (SD, range)**	85.7 (4.1, 54–100)	77.6 (10.8, 44–99)	65.7 (14.8, 16–89)	38.2 (22.7, 0–83)	190.097	< 0.001*
**IADL (SD, range)**	8.0 (0.1, 7–8)	7.5 (1.2, 3–8)	6.3 (1.3, 2–8)	2.1 (2.1, 0–8)	440.073	< 0.001*
**NPI-sum (SD, range)**	3.0 (4.1, 0–18)	5.8 (6.9, 0–29)	5.1 (6.4, 0–41)	10.4 (10.4, 0–63)	19.967	< 0.001***

CDR: Clinical Dementia Rating Scale; MCI: mild cognitive impairment; VMD: very mild dementia; N: number of participants; NMD-12Q: Normal-MCI-Dementia 12 Questionnaire; AD8: Ascertain Dementia 8; MMSE: Mini-Mental State Examination; MoCA: Montreal Cognitive Assessment; IADL: Instrumental Activities of Daily Living; NPI-sum: sum score of Neuropsychiatric Inventory.

* post hoc analysis showed CDR 0 < MCI < VMD < CDR≧1

** post hoc analysis showed CDR 0 = MCI > VMD = CDR≧1

*** post hoc analysis showed CDR 0 = MCI = VMD < CDR≧1.

**Fig 1:**
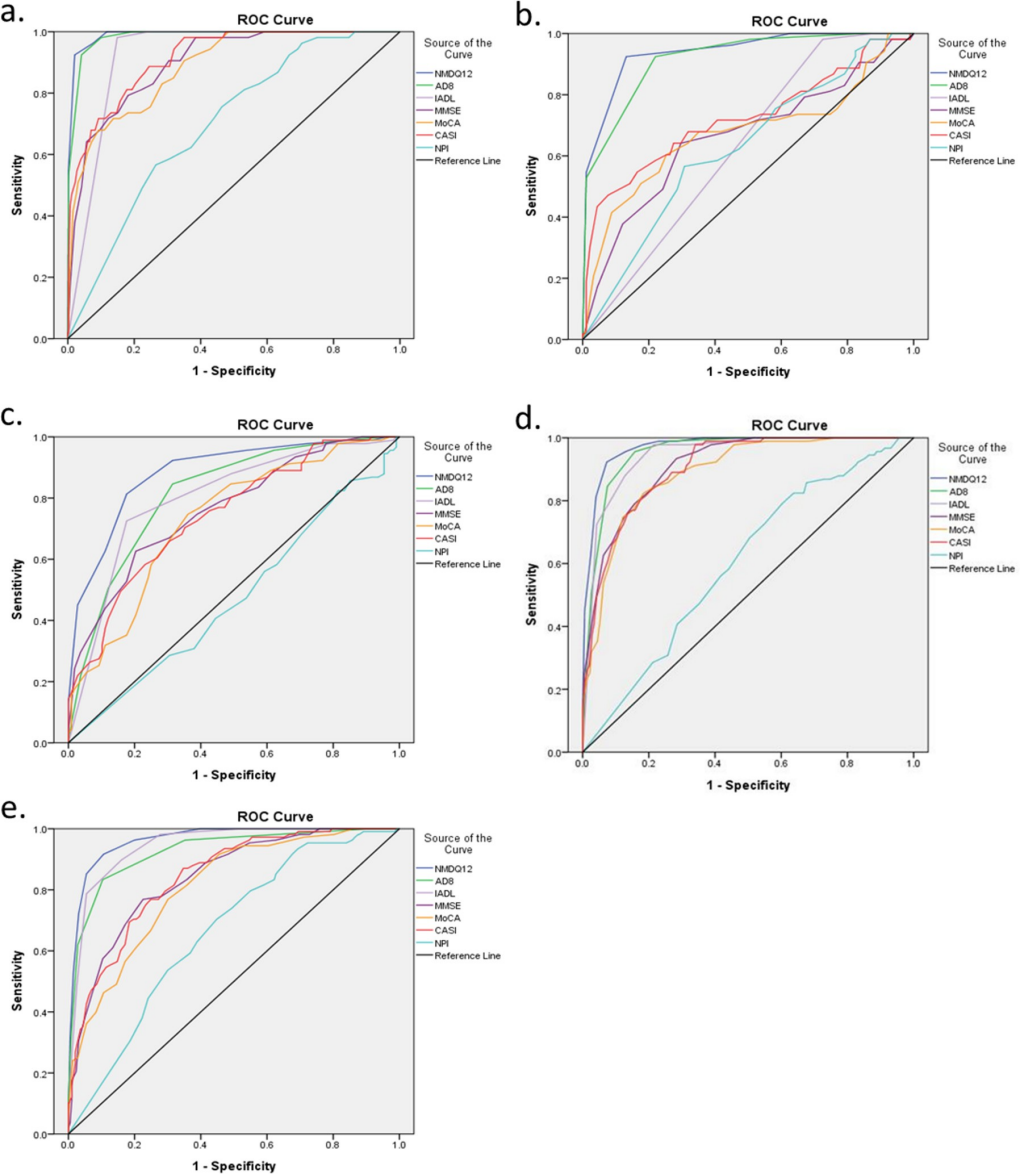
The receiver operating characteristic (ROC) curve analysis among all screening tools for different screening purposes. Fig 1A. NC vs all cognitive impairment; Fig 1B. NC vs MCI; Fig 1C. MCI vs VMD; Fig 1D. MCI vs all dementia; Fig 1E. VMD vs dementia with CDR≥1.

Table 2 compares the diagnostic accuracy of NMD-12, AD8, IADL, MMSE, MoCA, CASI and NPI for discriminating NC vs. cognitive impairment, NC vs. MCI, MCI vs. VMD, MCI vs. dementia, and VMD vs. dementia. As shown in Fig 1, NMD-12 consistently showed the highest diagnostic value in the ROC curve analysis among all screening tools for different screening purposes.

**Table 2:** Comparison of sensitivity, specificity, and area under curve (AUC), and cutoff score among all participants with different screening tools.

	NMD-12	AD8	IADL	MMSE	MoCA	CASI	NPI
**NC vs CI**							
**sensitivity**	0.98	0.96	0.98	0.79	0.72	0.81	0.73
**specificity**	0.93	0.93	0.85	0.82	0.86	0.82	0.57
**AUC**	0.99	0.98	0.92	0.90	0.89	0.92	0.70
**AUC 95%CI**	0.98–1.00	0.98–1.00	0.90–0.95	0.86–0.94	0l85-0.93	0.88–0.95	0.63–0.76
**cutoff**	1/2	1/2	8/7	23/22	18/17	76/75	2/3
**NC vs MCI**							
**sensitivity**	0.87	0.78	0.98	0.64	0.68	0.68	0.63
**specificity**	0.93	0.93	0.27	0.70	0.65	0.68	0.62
**AUC**	0.94	0.92	0.63	0.66	0.67	0.72	0.63
**AUC 95%CI**	0.90–0.98	0.87–0.96	0.54–0.72	0.57–0.76	0.57–0.77	0.63–0.82	0.53–0.72
**cutoff**	1/2	1/2	8/7	27/26	20/19	83/82	3/4
**MCI vs VMD**							
**sensitivity**	0.81	0.68	0.73	0.63	0.77	0.70	0.41
**specificity**	0.82	0.85	0.86	0.79	0.60	0.65	0.56
**AUC**	0.88	0.81	0.80	0.76	0.73	0.74	0.47
**AUC 95%CI**	0.84–0.93	0.76–0.87	0.74–0.86	0.70–0.83	0.66–0.80	0.68–0.81	0.39–0.55
**cutoff**	4/5	3/4	8/7	24/23	14/13	74/73	4/5
**MCI vs dementia**							
**sensitivity**	0.92	0.84	0.98	0.93	0.82	0.80	0.50
**specificity**	0.93	0.96	0.78	0.72	0.82	0.83	0.68
**AUC**	0.97	0.95	0.95	0.91	0.89	0.91	0.60
**AUC 95%CI**	0.96–0.98	0.94–0.97	0.93–0.97	0.89–0.94	0.86–0.92	0.88–0.93	0.54–0.66
**cutoff**	5/6	4/5	7/6	18/17	13/12	69/68	6/7
**VMD vs CDR≧1**							
**sensitivity**	0.95	0.90	0.90	0.77	0.77	0.69	0.61
**specificity**	0.85	0.83	0.84	0.77	0.70	0.82	0.63
**AUC**	0.96	0.93	0.95	0.84	0.81	0.84	0.66
**AUC 95%CI**	0.95–0.98	0.90–0.96	0.93–0.97	0.80–0.88	0.77–0.85	0.80–0.88	0.61–0.72
**cutoff**	8/9	5/6	5/4	17/16	8/7	62/61	5/6

NC: normal cognition; CI: cognitive impairment; MCI: mild cognitive impairment; VMD: very mild dementia; NMD-12: Normal-MCI-Dementia 12 questionnaire; AD8: Ascertain Dementia 8; MMSE: Mini-Mental State Examination; MoCA: Montreal Cognitive Assessment; IADL: Instrumental Activities of Daily Living; NPI: Neuropsychiatric Inventory.

## Discussion

In this study, we developed and validated a new, brief and accurate instrument, NMD-12, based on ML to discriminate NC from MCI as well as MCI from dementia. This instrument has a satisfactory high sensitivity (0.87) and specificity (0.92) for the screening of cognitive impairment from normal cognition. The practicality and interpretability of this new instrument were also confirmed by the experts in our clinical team.

The existing screening tools have a lot of limitations when being applied to populations with different cultures and different languages. We take the most widely used screening tools such as AD8 [1], Mini-Mental State Examination (MMSE) [6], and other tools for examples. First, AD8 is a brief informant interview for the screening of early cognitive change [1–5]. It is useful in most situations. However, a relatively low sensitivity [7] or low specificity [8] is noticed in some studies. Hence, some studies combined AD8 and other tools in one or more short cognitive tests and considered the combination to be more useful in detecting cognitive impairment than using the AD8 alone [2,5]. Second, AD8 is used to screen cognitively impaired individuals from normal population whereas studies seldom address the ability to differentiate MCI from dementia. It is relatively important because until now, there is only a pharmacological treatment for dementia but not for MCI. Third, the cutoff scores also vary across different countries or cultures. For example, the normal/impaired cutoff scores rise from 0/1 [2], 1/2 [1,3,5–8], 2/3 [4], to 3/4 [9]. Choosing a suitable cutoff score seems to be challenging. Additionally, identifying cutoff scores in cognitive screening test such as Mini-Mental State Examination (MMSE) [6], Montreal Cognitive Assessment (MoCA) [10,11], or Cognitive Abilities Screening Instrument (CASI) [12,13] are even more challenging for clinical applications. These three tools are also very commonly used screening instruments all over the world. However, MMSE, MoCA, and CASI are very sensitive to age and education level and multiple cutoff scores must be used for the diagnosis of cognitive impairment or dementia accordingly [11,13]. More importantly, a large variety of cutoff scores [10,11,13] was found in populations in undeveloped countries and areas where most of the elderly have low level of education, such as in Taiwan [13]. On the contrary, AD8 does not seem so sensitive to age or education level. Fourth, the information acquisition may be different from participant to patient or from the informant. For example, the ROC curve for the informant in a study using AD8 revealed that the AUCs (area under curve) were 0.89 (95% confidence interval, 0.86–0.93) vs 0.79 (95% confidence interval, 0.68–0.78) for the informants and participants, respectively [7].

Statistical machine learning, which performs a heuristic statistical search to find the regularities from a large dataset, has the potential to alleviate or eliminate the above problems and contribute to build a brief and accurate screening instrument. It should be noted that our method filtered out the redundant items and then built the optimal screening model using the ROC analysis. This combination of classic statistics by the ROC analysis and feature selection by information gain is different from other ML-derived methods which relied on classification algorithms, such as decision trees, Naïve Bayes, support vector machine and neural networks to build the screening models [21,22]. Our strategy has important advantages since 1) the non-linear classification methods are not as interpretable as our current method so the clinical practicality may be influenced; 2) our method can significantly reduce the scale of questionnaire; 3) more importantly, as shown in the following paragraphs, our method has superior accuracy to the current screening tools.

Our ML-derived method NMD-12 has relatively better diagnostic accuracy. Compared to the other screening tools, it is as effective as AD8 but superior to the other tools such as IADL, MMSE, MoCA, CASI, and NPI. It can also reliably discriminate MCI from dementia, with a high sensitivity (0.92) and specificity (0.93) which is higher than other tools such as AD8, IADL, MMSE, MoCA, CASI, and NPI. Moreover, NMD-12 also showed an acceptable accuracy with a relatively high sensitivity (0.81) and specificity (0.82) for the differentiation of MCI from VMD. Other screening tools showed less well results as demonstrated in Table 2. For the differentiation of dementia with CDR 0.5 (VMD) and CDR≧1, the NMD-12 also showed a relatively good result which is also superior to other screening tools (Table 2).

Noteworthy, our NMD-12 method is also clinically practical and interpretable. First, AD8 is superior to other cognitive (MMSE/MoCA/CASI) or activities of daily living instrument (IADL) for the screening of cognitive impairment from normal cognition regardless of the severity of cognitive impairment. However, for the screening of MCI/VMD from dementia, the AD8 is as ineffective or inefficient as other screening tools. Although there are items overlap or with the similarity between the NMD-12 and the AD8, the NMD-12 could demonstrate a superior screening value after the ML procedure. Additionally, the NMD-12 and the AD8 assess both changes in cognition and function, so it is expected that they are more accurate than scales that assess only cognition or IADLs. Second, none of MMSE, MoCA, and CASI meet the need for accurate differentiation of NC from MCI, or MCI from VMD. The cause is that all cognitive tests including MMSE, MoCA, or CASI are very sensitive to age and education level. Therefore, multiple cutoff scores must be used for the diagnosis of cognitive impairment or dementia according to ages and education levels. Third, IADL is useful for the differentiation of MCI and dementia. This finding is reasonable because significantly impaired instrumental activity of daily living is the key to the clinical diagnosis of dementia. However, IADL is not as widely applicable as our tool in discrimination of other stages of cognitive impairment. Fourth, although neuropsychiatric symptoms (NPS) demonstrated by NPI are well studied and the total score of NPI gets higher as the dementia severity progresses [23], the score also varies with different types of dementia [24]. Therefore, NPI is not quite useful for the diagnosis of different stages of cognitive impairment according to CDR staging system, probably due to a variety of the NPS that presents at different stages and for different types of individuals.

It should be noted that the elderly with a lower education level tend to have poor cognitive performance as compared with most of the cutoff scores reported from the previous studies. In our normal cognitive population with a mean education of 6.9 years, the mean performance of MoCA is only 21.3, and the cutoff score between NC and MCI in this study is as low as 18/17. Accordingly, MoCA is even more sensitive to education, and a typical cutoff score of 27/26 [10] will not be suitable for the population with relatively low education. Similar findings were reported in some other studies [11,25–27].

### Limitations

There are three limitations in this study. First, with the exception of a few individuals, the diagnosis of cognitive function is made mainly on the findings of CDR and cognitive screening tests (CASI/MMSE/MoCA), and no detailed neuropsychological test battery was used in most of the subjects. Second, our research was conducted in only three hospitals in Taiwan and our participants have relatively low education levels. Therefore, selection bias may arise, and a study in more medical centers with different languages or races is needed to further validate our method. Third, comparing the 95% CI of AUCs, the NMD-12 is superior to the AD8 only in distinguishing between MCI and VMD. Therefore, we are studying a longer screening instrument with significant content overlap with the NMD-12 for a better discrimination between different stages of dementia, especially in later stages of dementia.

## Conclusions

NMD-12 derived from machine learning is a simple and effective screening tool for discriminating NC, MCI, and dementia. Further studies should be warranted for assessing its role in the diagnosis and management of dementia.

## Supporting information

S1 Appendix Composition of the NMD-12 questionnaire. (DOCX)

## References

[1] Galvin JE, Roe CM, Xiong C, et al Validity and reliability of the AD8 informant interview in dementia. *Neurology* 2006;67(11):1942–48. 10.1212/01.wnl.0000247042.15547.eb 17159098

[2] Chin R, Ng A, Narasimhalu K, et al Utility of the AD8 as a self-rating tool for cognitive impairment in an Asian population. American Journal of Alzheimer's Disease & O*ther Dementias* *®* 2013;28(3):284–88. 10.1177/1533317513481090 PMC10852952 23493722

[3] Shaik MA, Xu X, Chan QL, et al The reliability and validity of the informant AD8 by comparison with a series of cognitive assessment tools in primary healthcare. International psychogeriatrics 2016;28(3):443–52. 10.1017/S1041610215001702 26489991

[4] Chan QL, Xu X, Shaik MA, et al Clinical utility of the informant AD8 as a dementia case finding instrument in primary healthcare. *Journal of Alzheimer's Disease* 2016;49(1):121–27. 10.3233/JAD-150390 26444776

[5] Yang L, Yan J, Jin X, et al Screening for Dementia in Older Adults: Comparison of Mini-Mental State Examination, Mini-Cog, Clock Drawing Test and AD8. PloS one 2016;11(12):e0168949 10.1371/journal.pone.0168949 28006822 PMC5179268

[6] Folstein MF. A practical method for grading the cognitive state of patients for the clinician. *J Psychiatr res* 1975;12:189–98. 1202204 10.1016/0022-3956(75)90026-6

[7] Ryu HJ, Kim H-J, Han S-H. Validity and reliability of the Korean version of the AD8 informant interview (K-AD8) in dementia. Alzheimer Disease & Associated Disorders 2009;23(4):371–76. 19561437 10.1097/WAD.0b013e31819e6881

[8] Meguro K, Kasai M, Nakamura K. Reliability and validity of the Japanese version of the AD8. *Nihon Ronen Igakkai zasshi Japanese journal of geriatrics* 2015;52(1):61–70. 10.3143/geriatrics.52.61 25786630

[9] Pardo CC, de la Vega Cotarelo R, Alcalde SL, et al Assessing the diagnostic accuracy (DA) of the Spanish version of the informant-based AD8 questionnaire. Neurología (English Edition) 2013;28(2):88–94. 10.1016/j.nrl.2012.03.012 PMC3485452 22652137

[10] Nasreddine ZS, Phillips NA, Bédirian V, et al The Montreal Cognitive Assessment, MoCA: a brief screening tool for mild cognitive impairment. *Journal of the American Geriatrics Society* 2005;53(4):695–99. 10.1111/j.1532-5415.2005.53221.x 15817019

[11] Chen KL, Xu Y, Chu AQ, et al Validation of the Chinese version of Montreal Cognitive Assessment basic for screening mild cognitive impairment. *Journal of the American Geriatrics Society* 2016;64(12) 10.1111/jgs.14530 27996103

[12] Teng EL, Hasegawa K, Homma A, et al The Cognitive Abilities Screening Instrument (CASI): a practical test for cross-cultural epidemiological studies of dementia. International Psychogeriatrics 1994;6(1):45–58. 8054493 10.1017/s1041610294001602

[13] Lin K-N, Wang P-N, Liu C-Y, et al Cutoff scores of the cognitive abilities screening instrument, Chinese version in screening of dementia. Dementia and geriatric cognitive disorders 2002;14(4):176–82. 10.1159/000066024 12411759

[14] McKhann GM, Knopman DS, Chertkow H, et al The diagnosis of dementia due to Alzheimer’s disease: Recommendations from the National Institute on Aging-Alzheimer’s Association workgroups on diagnostic guidelines for Alzheimer's disease. Alzheimer's & dementia 2011;7(3):263–69. 10.1016/j.jalz.2011.03.005 PMC3312024 21514250

[15] Morris JC. The Clinical Dementia Rating (CDR): current version and scoring rules. *Neurology* 1993. 10.1212/wnl.43.11.2412-a 8232972

[16] Albert MS, DeKosky ST, Dickson D, et al The diagnosis of mild cognitive impairment due to Alzheimer’s disease: Recommendations from the National Institute on Aging-Alzheimer’s Association workgroups on diagnostic guidelines for Alzheimer's disease. Alzheimer's & dementia 2011;7(3):270–79. 10.1016/j.jalz.2011.03.008 PMC3312027 21514249

[17] Lawton MP, Brody EM. Assessment of older people: Self-maintaining and instrumental activities of daily living. Gerontologist 1969;9:179–186. 5349366

[18] Cummings JL, Mega M, Gray K, et al The Neuropsychiatric Inventory comprehensive assessment of psychopathology in dementia. Neurology 1994;44(12):2308–08. 7991117 10.1212/wnl.44.12.2308

[19] Hall M, Frank E, Holmes G, Pfahringer B, Reutemann P, Witten IH. The WEKA data mining software: an update. ACM SIGKDD Explor Newsl 2009;11:10–18.

[20] Hall MA, Holmes G. Benchmarking attribute selection techniques for discrete class data mining. *IEEE Transactions on Knowledge and Data engineering* 2003;15(6):1437–47.

[21] Shankle WR, Datta P, Dillencourt M, et al Improving dementia screening tests with machine learning methods. Alzheimer's Research 1996;2(3)

[22] Chen R, Herskovits EH. Machine-learning techniques for building a diagnostic model for very mild dementia. Neuroimage 2010;52(1):234–44. 10.1016/j.neuroimage.2010.03.084 20382237 PMC2917811

[23] Siafarikas N, Selbaek G, Fladby T, et al Frequency and subgroups of neuropsychiatric symptoms in mild cognitive impairment and different stages of dementia in Alzheimer's disease. International Psychogeriatrics 2017:1–11. 10.1017/S1041610217001879 28927477

[24] Chiu P-Y, Tsai C-T, Chen P-K, et al Neuropsychiatric Symptoms in Parkinson’s Disease Dementia Are More Similar to Alzheimer’s Disease than Dementia with Lewy Bodies: A Case-Control Study. *PloS one* 2016;11(4):e0153989 10.1371/journal.pone.0153989 27101140 PMC4839640

[25] Carson N, Leach L, Murphy KJ. A re‐examination of Montreal Cognitive Assessment (MoCA) cutoff scores. *International Journal of Geriatric Psychiatry* 2017 10.1002/gps.4756 28731508

[26] Bosco A, Spano G, Caffò AO, et al Italians do it worse. Montreal Cognitive Assessment (MoCA) optimal cut-off scores for people with probable Alzheimer’s disease and with probable cognitive impairment. Aging Clinical and Experimental Research 2017:1–8. 10.1007/s40520-017-0727-6 28155182

[27] Zhou Sa, Zhu J, Zhang N, et al The influence of education on Chinese version of Montreal cognitive assessment in detecting amnesic mild cognitive impairment among older people in a Beijing rural community. *The Scientific World Journal* 2014;2014 10.1155/2014/689456 PMC4058117 24982978

