# Supplementary material for: NMD-12: A new machine-learning derived screening instrument to detect mild cognitive impairment and dementia
Source: PLoS One. 2019 Mar 8;14(3):e0213430. doi: 10.1371/journal.pone.0213430 (PMC6407752; doi:10.1371/journal.pone.0213430)
Supplement: S1 Appendix — (DOCX) [file pone.0213430.s001.docx]

**Appendix**

**Appendix 1. Composition of the NMD-12 Questionnaire.**

| 1 | 認知功能退化已經影響到日常生活，社交，與工作嗎? (例如獨立外出購物看病或處理事務等) | □ 不會 | □ 會 |
| --- | --- | --- | --- |
|  | Has cognitive impairment influence daily life, social networking and work? (For example, going out independently for shopping, seeing a doctor or working) | □ No | □ Yes |
| 2 | 會忘記正確的年份和月份嗎? | □ 不會 | □ 會 |
|  | Does he/she forget the correct year and month? | □ No | □ Yes |
| 3 | 記住約會的時間變得困難嗎? | □ 不會 | □ 會 |
|  | Is it difficult to remember the time of appointment? | □ No | □ Yes |
| 4 | 處理複雜的財務,例如上銀行, 繳費,開支票等變得困難嗎? | □ 不會 | □ 會 |
|  | Is it difficult to deal with complicated financing issues, such as going to bank, making payment or writing a check? | □ No | □ Yes |
| 5 | 認知功能(例如記憶，思考，判斷力)明顯地比以前退化嗎? | □ 不會 | □ 會 |
|  | Is cognitive function (such as memorization, thinking, judgment) significantly worse than before? | □ No | □ Yes |
| 6 | 例行的活動(例如散步、唸佛、拜拜、禱告、上教堂)或平常的嗜好興趣(例如跳土風舞、打牌、麻將、卡拉OK、與孫子女玩等)會不會比以前少了? | □ 不會 | □ 會 |
|  | Are regular activities (such as walking, reading, praying, going to church) or ordinary hobbies (such as dancing, playing cards, mahjong, karaoke, playing with grandchildren) less than before? | □ No | □ Yes |
| 7 | 自行服用藥物變得困難嗎? | □ 不會 | □ 會 |
|  | Is it difficult to take medicine by yourself? | □ No | □ Yes |
| 8 | 學習如何使用工具,設備變得困難嗎? | □ 不會 | □ 會 |
|  | Is it difficult to learn how to use tools or equipment? | □ No | □ Yes |
| 9 | 外出活動(搭車或騎/開車到目的地)變得困難嗎? | □ 不會 | □ 會 |
|  | Is it difficult to get out (ride or drive to destination)? | □ No | □ Yes |
| 10 | 金錢處理能力變差了嗎? | □ 不會 | □ 會 |
|  | Is it getting worse to manage money? | □ No | □ Yes |
| 11 | 工作能力或職業技術變差了嗎? | □ 不會 | □ 會 |
|  | Are working or professional skills getting worse? | □ No | □ Yes |
| 12 | 會常常忘記最近講過的話嗎? | □ 不會 | □ 會 |
|  | Do you often forget what you've talked about recently? | □ No | □ Yes |
